# Cu-doped ZnO nanoparticles and its application for the photocatalytic degradation of Rhodamine B

**DOI:** 10.1038/s41598-025-02432-x

**Published:** 2025-05-25

**Authors:** Guijuan Chen, Miao Yang, Beibei Tian, Jun Yao, Songlin Chen, Deming Li, Guojun Yuan

**Affiliations:** Anhui Vocational and Technical College, Collaborative Innovation Center for Chemistry and Life Sciences, Hefei, 230011 China

**Keywords:** Cu-doped ZnO, Rhodamine B, UV light irradiation, Pollution remediation, Environmental impact

## Abstract

Rhodamine B (RhB), a dye with high environmental pollution risks, necessitates effective degradation strategies for environmental protection. Photocatalytic degradation technology offers a viable solution to mitigate such pollution, with ZnO being extensively utilized in dye degradation due to its excellent ultraviolet (UV) photocatalytic performance. In this study, Cu-doped ZnO nanoparticles were successfully synthesized via a facile method. When the Cu doping level was 0.5%, the material exhibited remarkable photocatalytic activity under UV irradiation, achieving complete RhB degradation within 2 h (degradation efficiency up to 100%). The physical and chemical properties of the material were systematically analyzed using X-ray diffraction (XRD), scanning electron microscopy (SEM), and transmission electron microscopy (TEM). The results indicated that Cu incorporation did not alter the crystallinity of ZnO but significantly enhanced its light response capability. Based on the experimental results of UV-catalyzed RhB solution degradation, the photocatalytic mechanism of Cu-doped ZnO was thoroughly investigated, providing theoretical insights and technical support for the development of high-efficiency photocatalysts for dye degradation.

**Supplementary Information:**

The online version contains supplementary material available at 10.1038/s41598-025-02432-x.

## Introduction

Water pollution is a significant ecological issue that is anticipated to grow further in the future as a result of the substantial amount of industrial waste material discharged into natural bodies of water. Among the common methods for removing pollutants, Fenton process^1^ and photocatalysis are both relatively convenient approaches. Photocatalytic materials provide a promising and extensively studied approach to wastewater treatment through the photodegradation of pollutants (organic or inorganic) into simpler and non-toxic components^2^. Most dyes are made of compounds with strong chemical and physical properties, which have high solubility and stability in water. Long-term accumulation in nature poses risks to the environment and human health, and can lead to various hazards, including toxic effects and diminished light penetration in water^3,4^. However, dye pollution to water is still a big problem that has not been solved^5–9^. Among many textile dyes, Rhodamine B is a toxic synthetic dye with potential carcinogenicity, mutagenicity, and cardiotoxicity. Exposure to or ingestion of Rhodamine B can cause skin irritation, eye irritation, gastrointestinal discomfort, and respiratory distress, and may even lead to death^10,11^.

There are numerous traditional degradation methods for water pollutants, such as the anaerobic digestion method. Photocatalytic methods, however, have gained favor due to their inherent advantages, and their modification techniques have also intersected with other physical and chemical methods. For instance, adding compounds like persulfate, NaBH₄ (sodium borohydride), or peroximonosulfate can activate (catalyze) photocatalytic reactions, potentially enhancing the degradation efficiency. Moreover, photocatalytic technology has been applied in fields such as hydrogen production and ammonia synthesis^1,12–16^. The photocatalytic process using semiconductor nanoparticles such as TiO2, ZnO, Fe2O3 and ZnS is considered to be an effective technology for degradation of organic pollutants^17^. Among them, TiO2 and ZnO^18–20^ are the most easily found and commonly used semiconductor materials.ZnO is an excellent oxidant, used as a photocatalyst, because of its strong absorption of ultraviolet rays in the solar spectrum, so it has high efficiency.Compared with other catalysts, ZnO can produce H2O2 more efficiently^19,21^, which is suitable for the degradation of dyes in water. When ZnO materials are irradiated by UV light in water, a photocatalytic process occurs, in which valence band electrons are excited to conduction band. During this process, electrons (e^−^) are generated in the conduction band, and holes (h^+^) are formed in the valence band. The electrons subsequently undergo a reaction with oxygen molecules in water, resulting in the formation of superoxide anion radicals (O2^*^), while the holes react with hydroxyl ions in water, leading to the generation of hydroxyl radical compounds (OH^*^). The superoxide radicals further react with the electrons, and the H^+^ ions present in the water combine to form the compound H2O2. This H2O2 compound then engages with the electrons once again, resulting in the formation of the hydroxyl radical^22^. In reality, the performance of ZnO is insufficient for industrial applications. As a result, there is a need to enhance the optical properties of ZnO to enable it to produce electrons with UV light energy. One method of improving the performance of ZnO that has garnered considerable attention is doping transition metals. This modification approach introduces defects into the primary lattice, thereby generating new energy levels in the band gap^23^. Therefore, since metal ions play an important role in optical applications, it is reasonable to expect that the addition of transition metal ions to ZnO will support potential changes in their physical and optical properties. Many methods have been used to synthesize ZnO. Among chemical synthesis methods, ZnO synthesized by environmentally friendly route is popular due to its advantages of low cost, high toxicity and ecological friendliness^24–26^.In this work, Cu-doped ZnO composites were prepared using a simple solvothermal method with Zn(OAc)2·2H2O as the raw material^27,28^. The Cu-doped ZnO composites exhibited outstanding photocatalytic activity, which can be attributed to the reduced rate of electron–hole pair recombination. This is different from other reports^29,30^. Additionally, the photocatalytic performance of the synthesized catalyst in degrading Rhodamine B (RhB) dye in aqueous solutions was evaluated. The structure and properties of the photocatalysts were characterized using techniques such as energy dispersive X-ray spectroscopy (EDX), scanning electron microscopy (SEM), X-ray diffraction (XRD), specific surface area and porosity analysis (BET and BJH), and transmission electron microscopy (TEM). Finally, a proposed mechanism is presented to explain the influence of Cu doping on the photocatalytic activity of ZnO. Furthermore, the synergistic effect of Cu-doped ZnO on the degradation of RhB is investigated.

## Materials and methods

### Materials

All the chemicals were used without further purification. Zinc(II) acetate dihydrate (C4H10O6Zn, AR, 98.0%), Ammonium bicarbonate (NH_4_HCO_3_, AR), and Cupric(II) nitrate trihydrate (CuH6N2O9, AR, 98.0%) were purchased from Sinopharm Chemical Reagent Co., Ltd. Rhodamine B (C28H31ClN2O3, AR) was purchased from Adamas-beta.

### Catalyst preparation

Synthesis of nanometer zinc oxide samples by high temperature calcination. Zinc(II) acetate dihydrate (C4H10O6Zn) and Ammonium bicarbonate (CuH6N2O9) were dissolved in 100 ml deionized water, respectively. After standing for a period of time, the intermediate sediment is collected and washed three times with water and ethanol. The precipitates were placed in a 120 °C constant temperature drying box and kept warm for 3 h. Finally, the above-mentioned samples were ground and transferred to muffle furnace and calcined at 500 °C for 3 h to prepare nano-ZnO (ZnO NPs).

0.5%, 1%, 2%, 3% and 4% ammonium bicarbonate (*w*%) were added to certain amount of ZnO NPs, dissolved in ethanol and stirred in 80  °C constant temperature water bath until the ethanol evaporated completely. After solid grinding, copper-doped ZnO nanocrystalline was prepared by calcination in muffle furnace at 500 °C for 3 h (noted as 0.5% Cu/ZnO NPs, 1% Cu/ZnO NPs, 2% Cu/ZnO NPs, 3% Cu/ZnO NPs and 4% Cu/ZnO NPs).

### Characterizations

The powder X-ray diffraction (XRD) measurement was performed using a Cu K α radiation (γ= 0.1541 nm) on an Ultima IV X-ray diffractometer, with a voltage of 40 kV and a current of 40 mA. The 2 θ scans ranged from 20° to 70° at a scanning speed of 10° min^−1^. The surface morphology of the catalyst was observed using a Sirion field emission scanning electron microscope (FESEM, TTR-III). N_2_ physisorption analysis was conducted based on the Brunauer-Emmett-Teller (BET, ASAP 2020 HD88) equation, while pore distributions were obtained using the Barrett-Joyner-Halenda (BJH, ASAP 2020 HD88) method. Transmission electron microscopy (TEM) was performed on a FEI-G20-2010 electron microscope operating at an acceleration voltage of 200 kV. XPS measurements were carried out using a Thermo ESCALAB 250Xi instrument. UV-vis DRS and EPR were tested respectively using Lambda 365 and P820-20EPR equipment.

### Catalytic activity testing

Cu/ZnO NPs were added into a beaker containing the prepared RhB solution and stirred in a dark environment for 30 min for photocatalytic reaction. For light reaction, the reaction solution is placed 15 cm away from the xenon lamp for 2 h. After the light was turned on, the reaction solution was stirred by magnetic agitator at a speed of 580 r/min, so that the Cu/ZnO NPs in the reaction solution could be fully dispersed in each liquid layer. In the process of reaction, appropriate amount of solution should be extracted regularly and quantitatively for detection. The measured solution is packed with a centrifuge tube and then placed in a centrifuge. After covering the centrifuge, set the centrifuge speed and rotation time to 12,000 rmp and 8 min respectively, and start the switch to centrifuge. Finally, the preheated ultraviolet–visible phototypesetter was debugged and its wavelength was set at 552 nm. The centrifuged degraded solution was carefully removed from the centrifuge tube.The concentration of the dye in the solution was measured by analyzing the RhB absorption intensity using a UV spectrometer and a dish to quantify the absorbance. Degradation rate H (%) can be calculated by the formula H = (B0 − B)/B0 × 100% (B0 is the absorbance before the reaction of the degraded solution, and B is the absorbance after the degradation of the degraded solution).

## Results and discussion

**Fig. 1:**
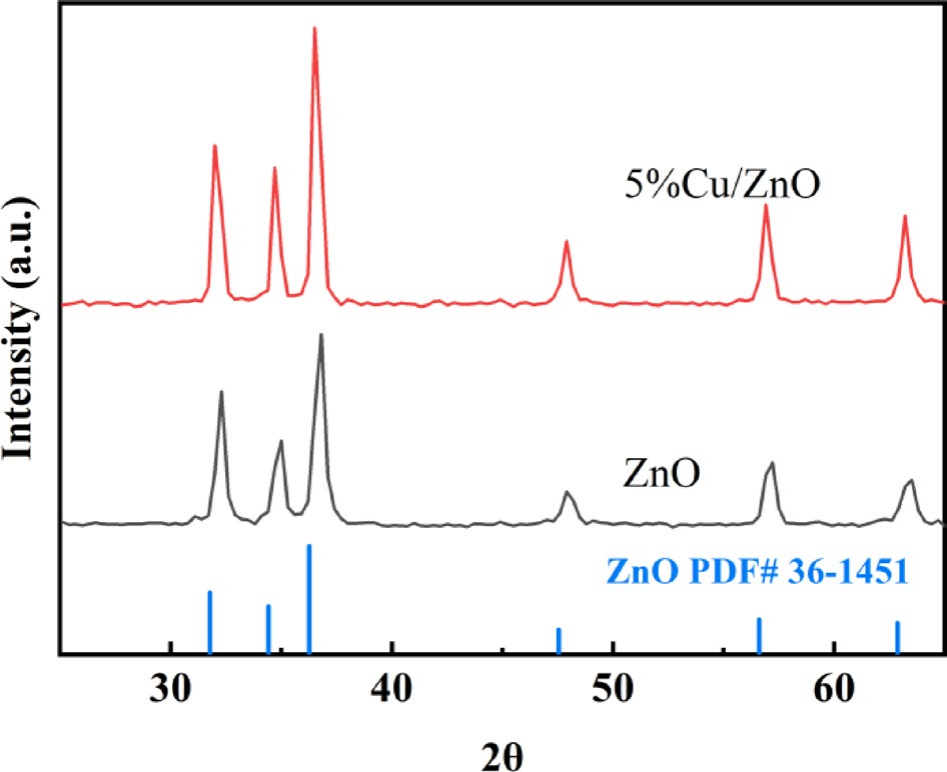
The XRD patterns of ZnO and 0.5% Cu/ZnO sample.

As an independent confirmation of the crystal structure of our samples, we performed X-ray diffraction analysis of the sample and the corresponding XRD patterns are shown in Fig. 1(Blank Line). The results show that there are many sharp characteristic peaks, which correspond to (100), (002), (101), (102), (110), (103) crystal planes of ZnO, respectively. The related peaks and all diffraction patterns can be labeled as wurtzite hexagonal phase of ZnO (JCPDS 36–1451). Further, no other impurity peaks are detected after Cu-doped, suggesting ZnO has not undergone structural changes as a consequence of Cu-doped. The results show that the doping of Cu has no effect on the microstructure of ZnO^31–33^. At the same time, the crystal type of catalyst samples did not change before and after doping, indicating that the samples were stable^34^.

**Fig. 2:**
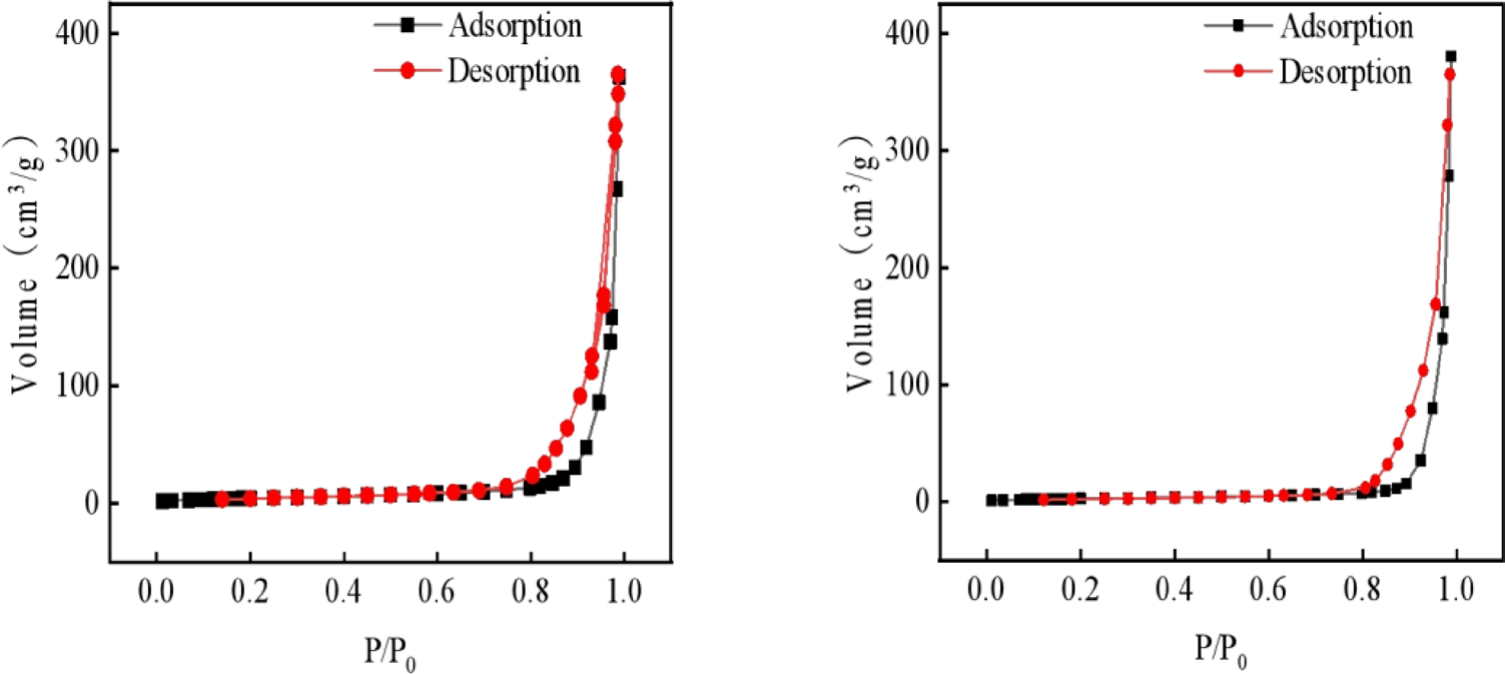
The N_2_ adsorption–desorption isothermal curves of ZnO and 0.5% Cu/ZnO samples.

In order to investigate the effect of Cu doping on the surface structure of ZnO, BET analysis was carried out on NPs and ZnO without Cu doping. The N2 adsorption–desorption curves of ZnO catalysts and 0.5% Cu/ZnO NPs have obvious H4 hysteresis ring, which is a typical type IV isotherm. As shown in Fig. 1(Red Line), the adsorption increases rapidly in the high pressure region (p/p0 = 0.8–1.0), indicating that both ZnO without Cu and 0.5% Cu/ZnO NPs catalysts have macroporous or mesoporous surface structures. The catalytic performance of the catalyst is related to its specific surface area and pore size. The large specific surface area and the small pore structure can provide more active sites for the photocatalytic reaction and effectively improve the photocatalytic performance of the material. Table 1 shows that the specific surface area of 0.5% Cu/ZnO NPs is twice as large as that of ZnO without Cu. The increase of the specific surface area provides more possibility for improving the adsorption and catalytic performance of the catalyst. The BET results in Fig. 2 show that the specific surface area of Cu ions is significantly increased after doping, which helps to expose more active sites.

**Fig. 3:**
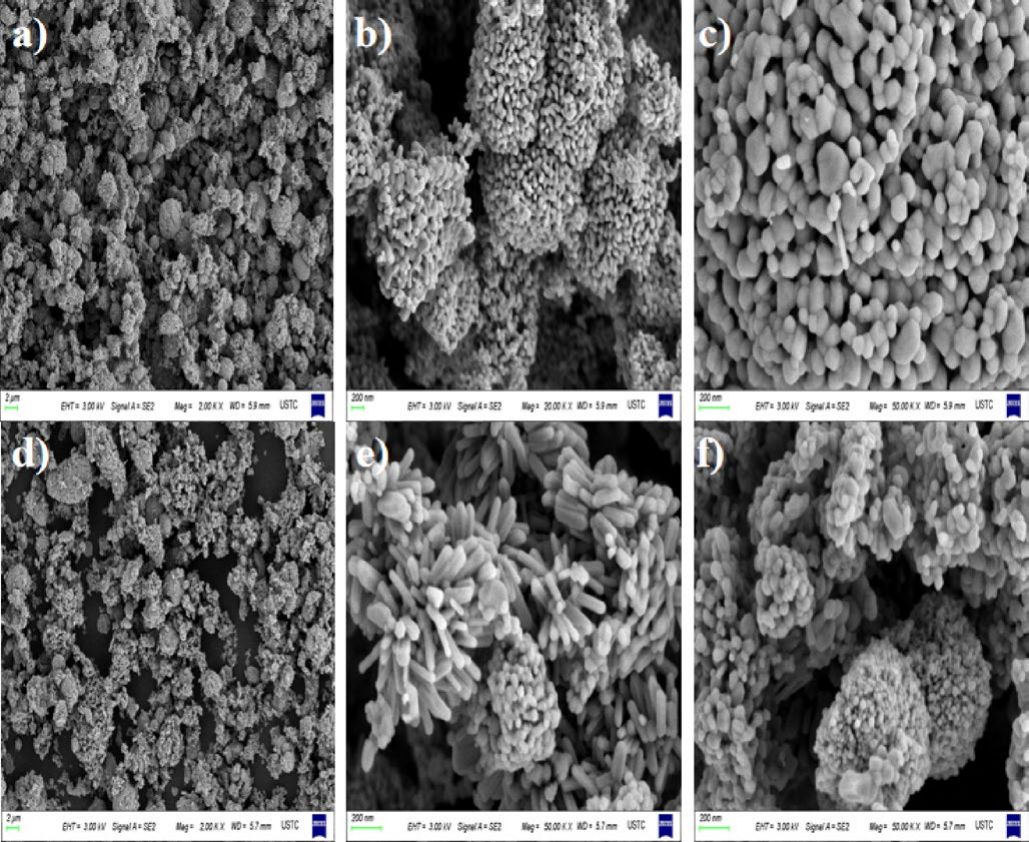
SEM images of ZnO and 0.5% Cu/ZnO samples (**a**–**c** is the SEM of ZnO catalyst, **d**–**f** is the SEM of 0.5% Cu/ZnO catalyst).

As illustrated in Fig. 3, scanning electron microscopy (SEM) images of pure ZnO are displayed in panels 3a, 3b, and 3c, while SEM images of ZnO samples doped with 0.5% copper, prepared under identical calcination conditions, are presented in panels 3d, 3e, and 3f. The SEM analysis reveals that the particle size of undoped ZnO is approximately 50 nm, with some particles exhibiting clustering behavior that is notably pronounced. In contrast, the copper-doped ZnO samples (Fig. 3d–f) exhibit smaller particle sizes and reduced clustering, with the particles adopting a characteristic hexagonal wurtzite structure. This morphological transformation is hypothesized to stem from the incorporation of copper ions into the ZnO crystal lattice, which lowers the system’s energy state. Additionally, the concentration gradient of copper ions between the interior and exterior of the crystal grains may drive diffusion-induced segregation along grain boundaries, thereby impeding further crystal growth^35,36^. Because of the irregular morphology of the catalyst, there was no significant change before and after the reaction, so the conclusions of relevant reports are consistent^37^. From the TEM image in Fig. S1, we can observe the microstructure of ZnO. Among them, d = 0.43 A corresponds to the lattice stripe width of the high-exposure crystal plane of ZnO^38,39^.

**Fig. 4:**
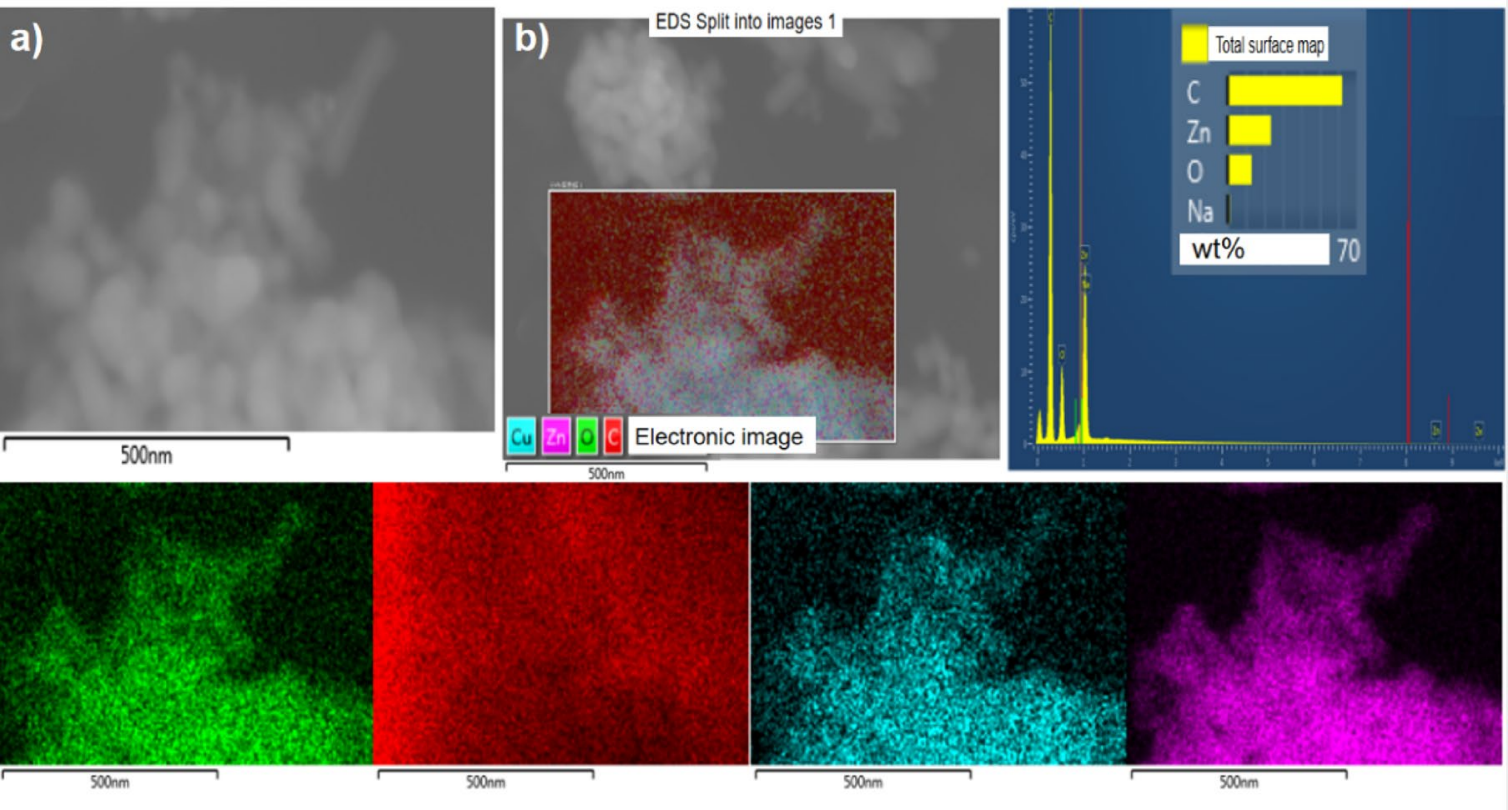
Energy spectrum analysis of 0.5% Cu/ZnO sample.

In addition, EDS testing was conducted on the samples to investigate their elemental distribution. According to the results (Fig. 4), it can be observed that copper (Cu), zinc (Zn), and oxygen (O) elements are distributed throughout the entire sample area. This further confirms the successful preparation of Cu/ZnO photocatalysts^40^.

**Fig. 5:**
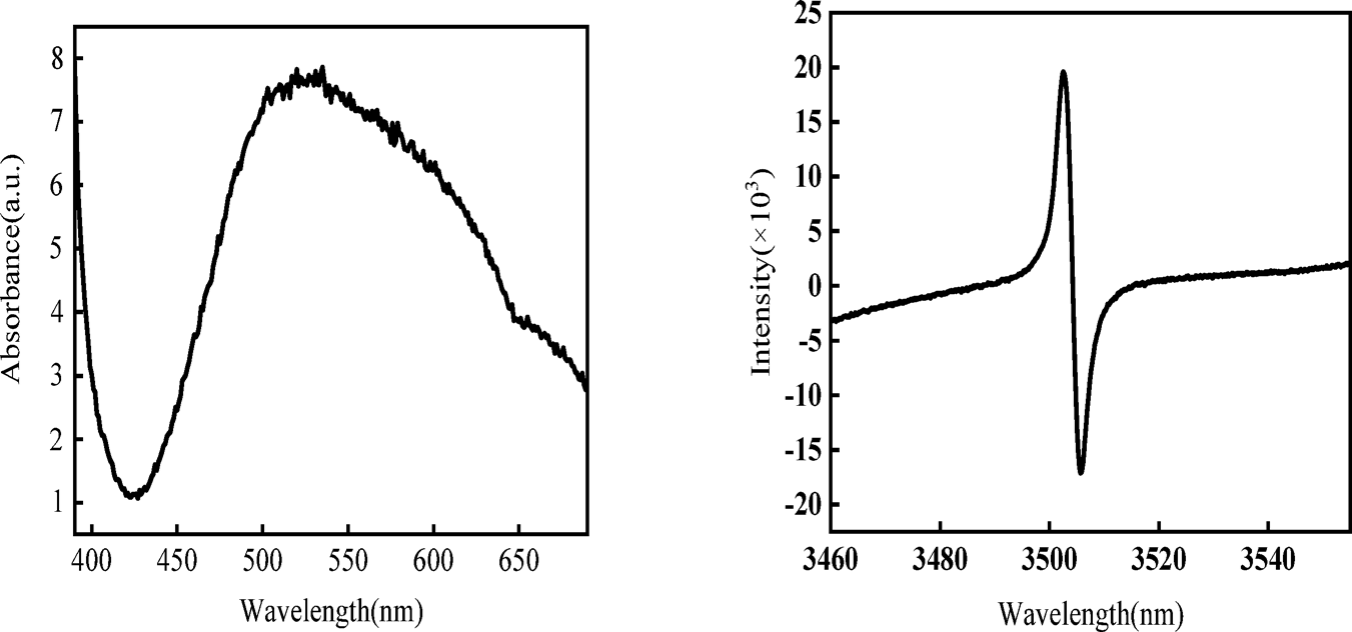
UV absorption spectrum of 0.5% Cu/ZnO at 369 nm (**a**) and EPR spectrum of 0.5% Cu/ZnO (**b**).

The fluorescence spectra of the 0.5% Cu/ZnO catalyst were acquired under single-photon excitation at a wavelength of 369 nm. As depicted in Fig. 5a, a distinct peak is observed near 470 nm. Upon reviewing the literature, it is noted that pure ZnO typically exhibits fluorescence peaks around 430 nm and 470 nm. The peak at approximately 430 nm is attributed to zinc vacancies, as highlighted in Fig. 5a. However, in the current study, only the 470 nm peak is observed, which corresponds to the green emission peak resulting from a radiative recombination process. This green emission peak originates from deep-level defect radiations, specifically the recombination of photogenerated holes and singly ionized electrons on oxygen vacancies during photocatalysis. The absence of the 430 nm peak, indicative of zinc vacancies, may be attributed to copper doping, although this does not impede the recombination of photogenerated holes and singly ionized electrons localized on oxygen vacancies during the photocatalytic process. Notably, the defect peak in the Cu-doped samples displays significant intensity, comparable to that of excitonic emission, which could be ascribed to the increased defect density induced by copper doping^41^. Additionally, the waveform variations around G = 3600 in Fig. 5b further corroborate the presence of vacancies within the sample. From Fig. S5, we conducted XPS analysis on the samples. The results revealed that Cu and O elements primarily exist in the + 2 and − 2 oxidation states, respectively. However, there is a noticeable tendency for Cu to transition towards the + 1 oxidation state, indicating that the doping process has induced a certain level of activity in the metal element.

**Fig. 6:**
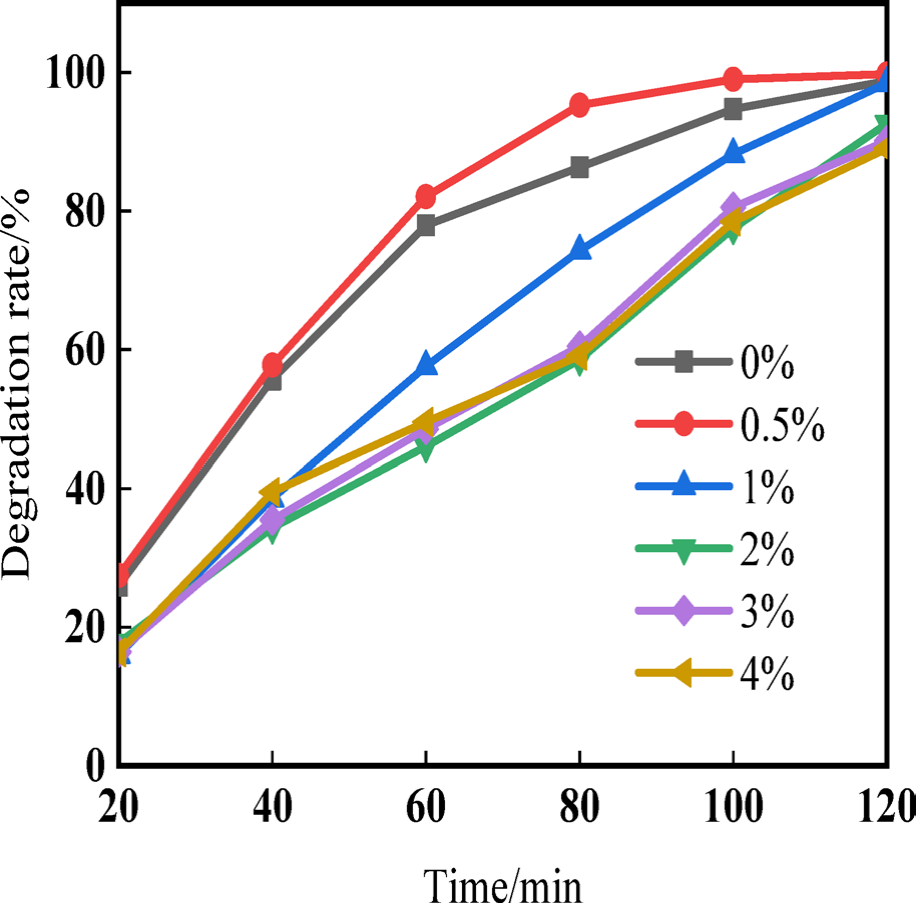
Photocatalytic degradation efficiency of Rhodamine B (RhB) under UV irradiation using ZnO nanoparticles with different Cu doping concentrations (0–4 wt%).

Figure 6 shows the degradation rate curves of different Cu-doped ZnO samples after calcination at 400 °C for 6 h, using Rhodamine B solution. According to the experimental data on the degradation of Rhodamine B by photocatalysts, it can be observed that after 120 min of UV irradiation, almost complete degradation of Rhodamine B is achieved with a copper doping level of 0.5%. Increasing the copper doping level leads to fluctuating changes in the final degradation rate of the solution, with a gradual decrease in the degradation rate. The analysis suggests that this may be due to the absorption of electrons generated during the photocatalytic process by copper ions. As a result, the probability of collisions between electrons and holes generated during the photocatalytic process decreases over time, while the increasing number of remaining holes accelerates the degradation of the solution. With the slow increase in Cu-doped level, a layer of Cu^+^ will cover the surface of nano ZnO. During UV irradiation, the amount of light absorbed by ZnO decreases, resulting in a natural decrease in its catalytic activity^42,43^. From Fig. 6, it can be concluded that ZnO exhibits good UV degradation performance towards Rhodamine B solution, and moderate Cu doping significantly enhances the degradation efficiency of ZnO towards Rhodamine B solution. The composite photocatalyst material, 0.5% Cu/ZnO, exhibits the highest photocatalytic activity.

**Fig. 7:**
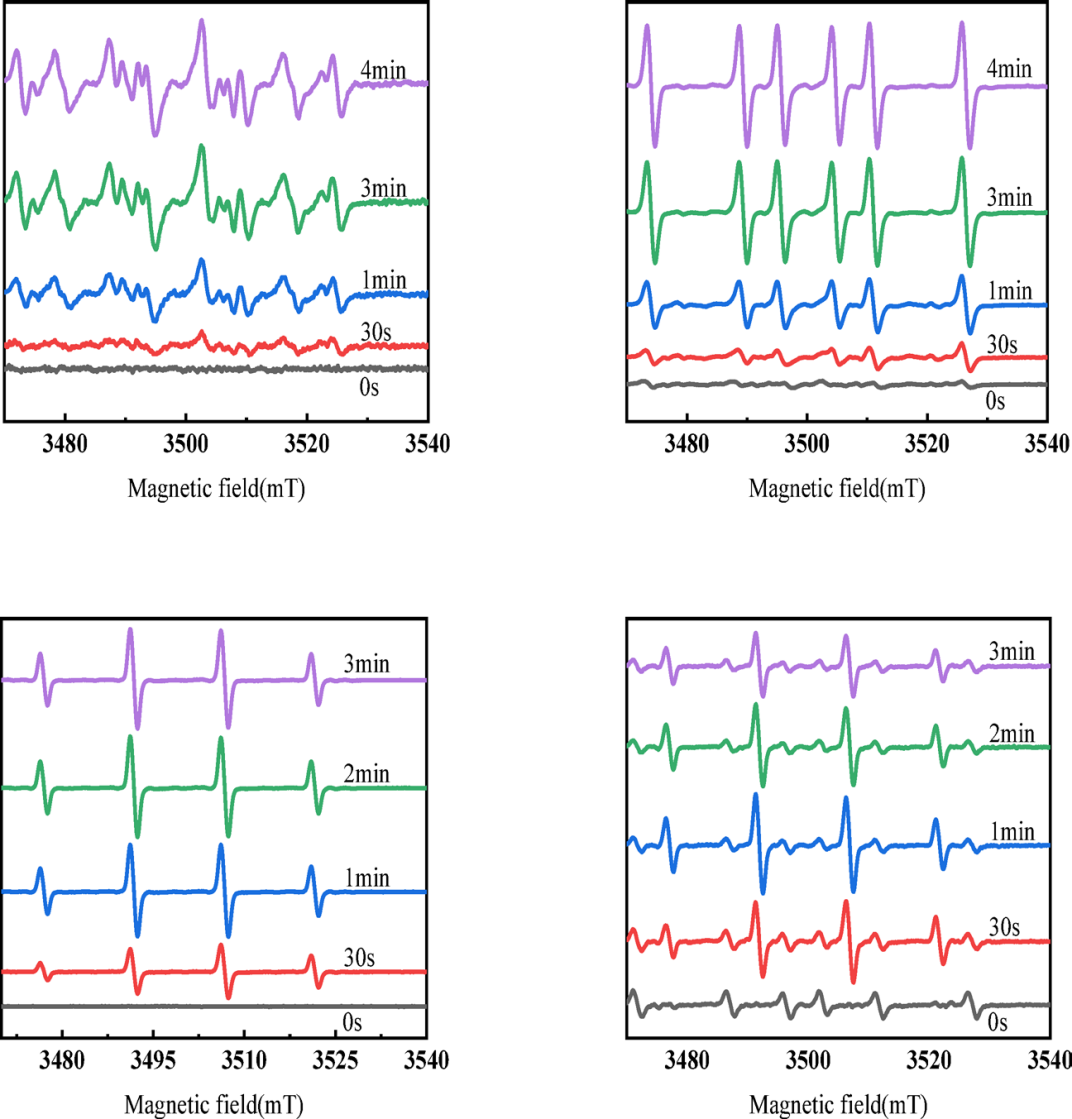
Electron paramagnetic resonance (EPR) spectra of reactive oxygen species generated during photocatalytic degradation: (**a**) Superoxide radical (·O₂⁻) signals from pure ZnO; (**b**) Enhanced ·O₂⁻ signals from 0.5% Cu-doped ZnO; (**c**) Hydroxyl radical (·OH) signals from pure ZnO; (**d**) Enhanced ·OH signals from 0.5% Cu-doped ZnO.

According to Fig. 7, under UV irradiation, a large number of holes are easier to produce, helping to provide more catalytic sites^44^. ZnO’s valence band is excited by absorbing the energy of photons, which generates excitonic electrons (e^−^), which will transition to the conduction band of ZnO and leave behind a hole (h^+^) $(ZnO - \to ^{hv} ZnO({\text{e}}^{ - } + {\text{h}}^{ + } ))$in the valence band. The resulting band gap energy facilitates the photocatalytic reaction^45,46^, the photocatalytic degradation of Cu doped ZnO is shown in Fig. S2^47–51^. Relevant reports indicate that there are studies on the impact of different pH values on degradation^52^. In our laboratory, reactions are conducted under mild pH conditions. The excitonic electrons and holes can then transfer to the surface of the ZnO crystal or to the Cu^2+^ surface through the ZnO, where they respectively react with O2 and H2O or OH^−^ in the solution to produce photocatalytically active ·O2^−^ and ·OH free radicals. The reaction equations are as follows^53–55^:

1 $${\text{O}}_{{2}} + {\text{e}}^{ - } \to \cdot{\text{O}}_{2}^{ - }$$

2 $${\text{H}}_{{2}} {\text{O}} + {\text{h}}^{ + } \to \cdot{\text{OH}} + {\text{H}}^{ + }$$

3 $${\text{OH}}^{ - } + {\text{h}}^{ + } \to \cdot{\text{OH}}$$

In the process of Cu-doping ZnO, Cu^+^ are ultimately anchored onto the surface of the ZnO crystal. Due to the strong oxygen adsorption capability of Cu^+^ (which may form under certain conditions), there is an increase in ·O_2_^−^ content near the ZnO crystal. This augmented ·O_2_^−^ presence plays a crucial role in photocatalytic processes: on one hand, it promotes the consumption of photogenerated excitonic electrons (e^−^), thereby effectively reducing the recombination rate of electron–hole (e^− ^− h^+^) pairs within the ZnO crystal; on the other hand, it enhances the generation of superoxide radicals (·O_2_^−^) and hydroxyl radicals (·OH), which are highly active and contribute to improved photocatalytic activity of the ZnO catalyst. However, when the concentration of Cu^+^ (C_(Cu+)_) exceeds 0.5%, the surface coverage of Cu^+^ on the ZnO crystal gradually increases, which paradoxically leads to changes in the ZnO crystal’s microstructure that ultimately reduce the total specific surface area available for light absorption. Consequently, the number of photogenerated excitonic electron–hole pairs decreases, resulting in a diminished synthesis of ·O_2_- and ·OH free radicals and a corresponding reduction in the photocatalytic degradation efficiency of the ZnO material. Moreover, under high doping concentrations, slight distortions occur in the ZnO crystal lattice, giving rise to an increased number of defects such as oxygen vacancies (V_O_) and zinc vacancies (V_Zn_)^56^. These defects can act as new recombination centers for electron–hole pairs (e^− ^− h^+^), further compromising the photocatalytic activity of ZnO^57–61^.

**Fig. 8:**
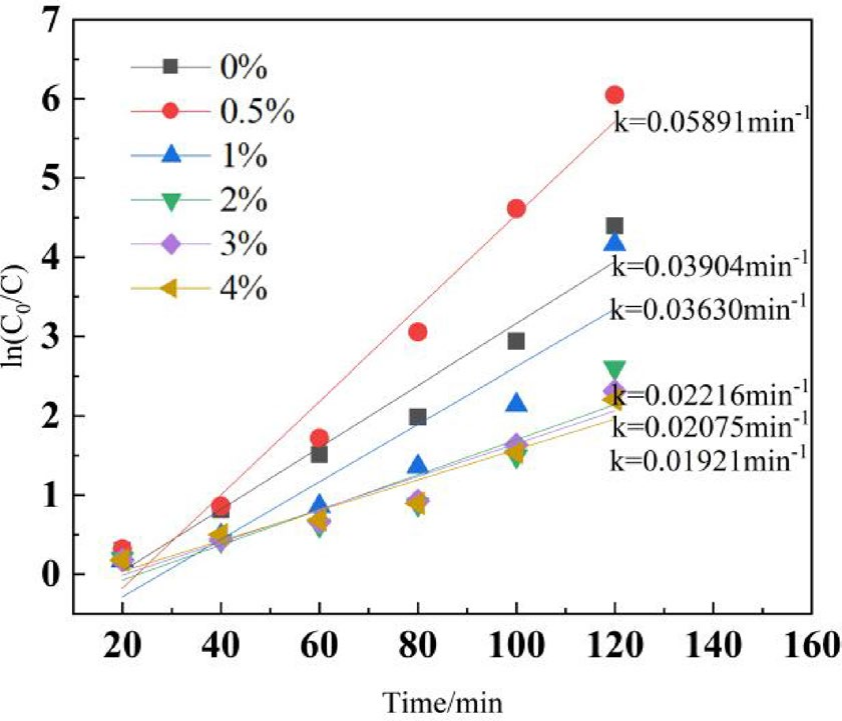
Kinetic curves of ZnO photodegradation with different Cu-doped.

Figure 8 illustrates the kinetic curve depicting the photocatalytic degradation of Rhodamine B using Cu/ZnO nanoparticles. A strong linear correlation is evident between ln(C0/Ct) and time (t) across various Cu-doped zinc oxide photocatalysts. Notably, the Cu-doped zinc oxide sample with a 0.5% mass fraction demonstrates the highest reaction rate constant (k), valued at 0.05891 min⁻^1^. Supplementary figures (Figs. S3 and S4) further reveal that, after a two-hour reaction period, both the degradation rate of Rhodamine B and its degradation kinetic constants exhibit optimal performance for the 0.5% Cu-doped sample. These findings align closely with those reported in the literature^62^.

## Conclusion

In this research, ZnO nanoparticles doped with varying concentrations of Cu were synthesized through a straightforward method. The results demonstrated that the photocatalytic degradation of Rhodamine B (RhB) was most efficient when the Cu content was 0.5%. The physicochemical properties of the synthesized samples were thoroughly characterized using techniques such as X-ray diffraction (XRD), X-ray photoelectron spectroscopy (XPS), scanning electron microscopy (SEM), and transmission electron microscopy (TEM).

The findings revealed that Cu doping did not affect the crystallinity of ZnO but significantly enhanced its photoresponse. Additionally, Cu-doped ZnO exhibited an increased specific surface area, which contributed to its enhanced photocatalytic performance. The degradation of RhB solution was notably more effective under ultraviolet (UV) irradiation. The proposed mechanism for RhB degradation by Cu-doped ZnO under UV irradiation involves the adsorption of Cu+ ions on the surface of ZnO crystals. This adsorption facilitates the consumption of photogenerated exciton electrons (e^−^), thereby effectively reducing the recombination of electron-hole (e^−^-h^+^) pairs within the ZnO crystals. Furthermore, the photocatalytic activity of ZnO was enhanced by the increased generation of reactive oxygen species, such as superoxide radicals (·O_2_^−^) and hydroxyl radicals (·OH). At a Cu/ZnO concentration of 0.5%, the ZnO crystal structure experienced slight distortion, which optimized its photocatalytic efficiency. However, as the Cu ion concentration increased, more defects were introduced into the crystal lattice, creating new recombination centers for electron-hole pairs. This phenomenon led to a reduction in the photocatalytic activity of ZnO, highlighting the importance of optimal doping levels for achieving maximum photocatalytic performance^63^.

## Electronic supplementary material

Below is the link to the electronic supplementary material.

Supplementary Material 1

## Data Availability

All data generated or analysed during this study are included in this published article.
