# Supplementary material for: Cu-doped ZnO nanoparticles and its application for the photocatalytic degradation of Rhodamine B
Source: Sci Rep. 2025 May 25;15:18246. doi: 10.1038/s41598-025-02432-x (PMC12104330; doi:10.1038/s41598-025-02432-x)
Supplement: Supplementary file 1 — Supplementary Material 1 [file 41598_2025_2432_MOESM1_ESM.docx]

**Cu-doped ZnO nanoparticles and its application for the**

**photocatalytic degradation of Rhodamine B**

Guijuan Chen1**#**, Miao Yang1**#**, Beibei Tian1, Jun Yao1^*^,Songlin Chen1^*^, Deming Li1^*^, Guojun Yuan1^*^

1 Anhui Vocational And Technical College, Hefei 230011, China

*Address correspondence to E-mail:165378341@qq.com (G.J. Yuan), [36369806@qq.com](mailto:36369806@qq.com) (D. M.Li), [64279445@qq.com (S.L](mailto:johncow2002@163.com).Chen), [johncow2002@163.com](mailto:johncow2002@163.com) (.J. Yao)

#contributed equally to this work.


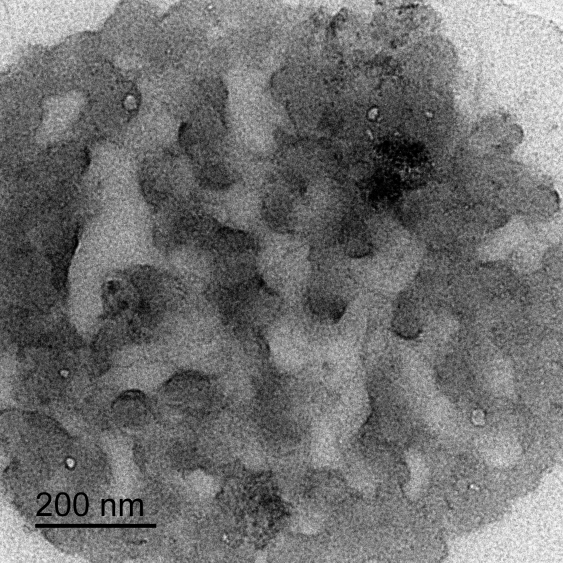

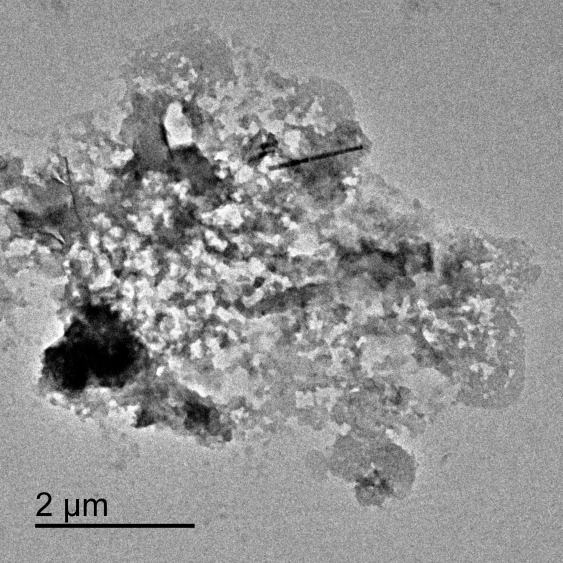


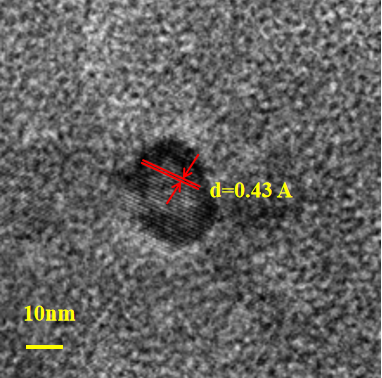

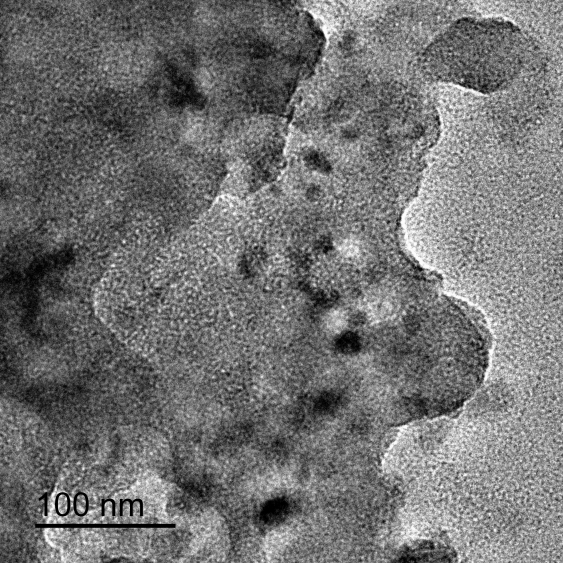


Fig. S1 TEM images of 0.5% Cu/ZnO samples


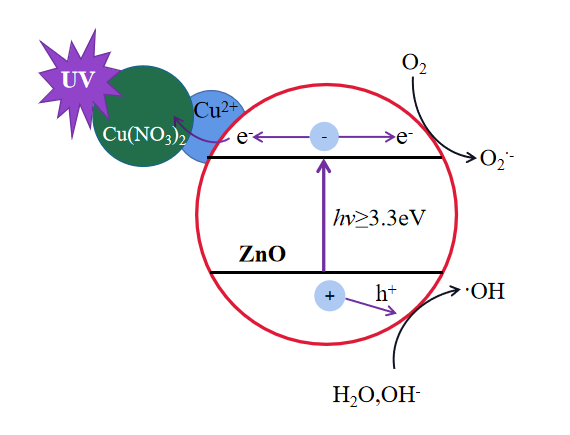


Fig. S2 Diagram of photocatalytic mechanism of ultraviolet irradiation


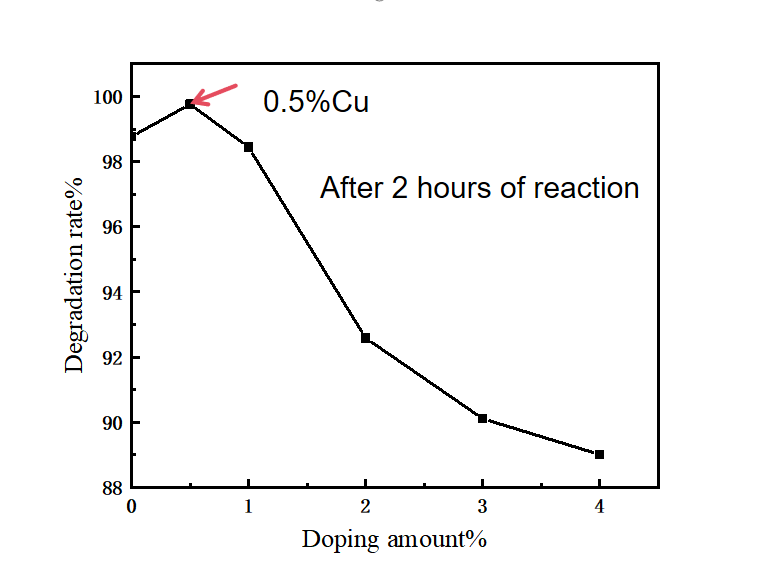


Fig. S3 .Changes in the degradation rate of Rhodamine B by copper with different doping levels


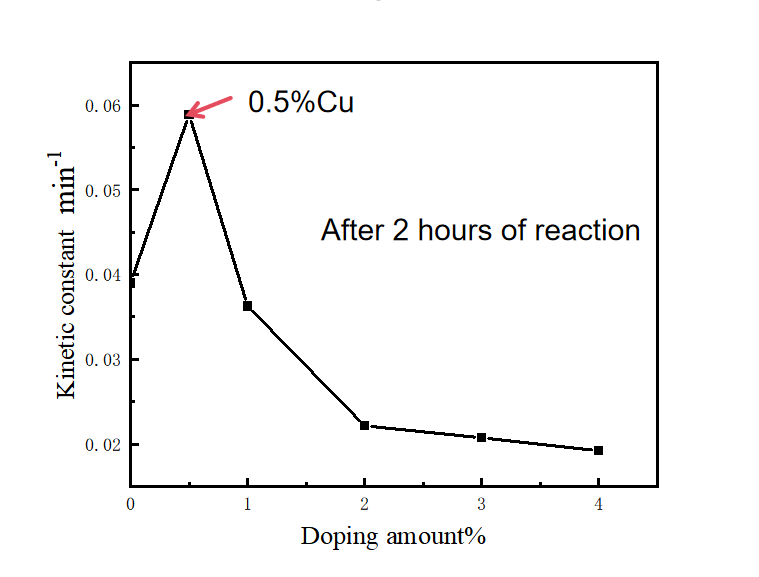


Fig. S4.Degradation kinetic constants of Rhodamine B by copper with different doping levels

Fig. S5 Detailed XPS spectra of the catalyst, Cu 2P and O 1s
